# Characterization of Au and Bimetallic PtAu Nanoparticles on PDDA-Graphene Sheets as Electrocatalysts for Formic Acid Oxidation

**DOI:** 10.1186/s11671-015-1071-4

**Published:** 2015-09-16

**Authors:** Tung-Yuan Yung, Ting-Yu Liu, Li-Ying Huang, Kuan-Syun Wang, Huei-Ming Tzou, Po-Tuan Chen, Chi-Yang Chao, Ling-Kang Liu

**Affiliations:** Department of Physics, National Central University, Jhongli, Taoyuan, 320 Taiwan; Molecular Science and Technology, Taiwan International Graduate Program/Institute of Chemistry, Academia Sinica, Taipei, 115 Taiwan; Nuclear Fuels and Materials Division, Institute of Nuclear Energy Research, Lontan, Taoyuan, 325 Taiwan; Department of Materials Engineering, Ming Chi University of Technology, New Taipei City, 24301 Taiwan; Department of Materials Science and Engineering, National Taiwan University of Science and Technology, Taipei, 106 Taiwan; Center for Condensed Matter Sciences, National Taiwan University, Taipei, 106 Taiwan; Materials Science and Engineering, National Taiwan University, Taipei, 106 Taiwan

**Keywords:** Graphene, PDDA, Au nanoparticle, Bimetallic PtAu nanoparticle, Formic acid oxidation

## Abstract

Nanocomposite materials of the Au nanoparticles (Au/PDDA-G) and the bimetallic PtAu nanoparticles on poly-(diallyldimethylammonium chloride) (PDDA)-modified graphene sheets (PtAu/PDDA-G) were prepared with hydrothermal method at 90 °C for 24 h. The composite materials Au/PDDA-G and PtAu/PDDA-G were evaluated by transmission electron microscopy (TEM), X-ray diffraction (XRD), X-ray photoelectron spectroscopy (XPS), and thermogravimetric analysis (TGA) for exploring the structural characterization for the electrochemical catalysis. According to TEM results, the diameter of Au and bimetallic PtAu nanoparticles is about 20–50 and 5–10 nm, respectively. X-ray diffraction (XRD) results indicate that both of PtAu and Au nanoparticles exhibit the crystalline plane of (111), (200), (210), and (311). Furthermore, XRD data also show the 2°–3° difference between pristine graphene sheets and the PDDA-modified graphene sheets. For the catalytic activity tests of Au/PDDA-G and PtAu/PDDA-G, the mixture of 0.5 M aqueous H_2_SO_4_ and 0.5 M aqueous formic acid was used as model to evaluate the electrochemical characterizations. The catalytic activities of the novel bimetallic PtAu/graphene electrocatalyst would be anticipated to be superior to the previous electrocatalyst of the cubic Pt/graphene.

## Background

Formic acid and methanol are potential in the conversions between chemical energy and electric energy as the chemical fuels. During earlier investigations, researchers have dismissed formic acid as a practical fuel because of the high overpotential evidenced by experiments, indicative that the reaction starting from formic acid appeared to be too difficult. The pristine Pt-based electrocatalysts have lowered the chemical barriers for conversion. Therefore, the bimetallic Pt-based nanomaterials were introduced as anode catalysts for methanol and formic acid oxidation [1–7]. Furthermore, Pt-M alloy and bimetallic core-shell nanoparticles promised for electrocatalytic reactions have been developed [5–7], which could enhance the electrocatalytic abilities by high CO-tolerance capability during the electrocatalysis. As a one-carbon-containing molecule similar to methanol, formic acid is a suitable organic molecule to be used directly in fuel cells, removing the need of complicated catalytic reforming. With a boiling point of 100.8 °C, formic acid is a liquid at standard temperature and pressure. Formic acid is accordingly much easier and much safer than hydrogen and methanol in energy storage because it does not need the high pressure or the low temperature operation. The efficiency of formic acid fuel cells can be engineered to a much higher level than that of methanol in addressing the crossover issue with the polymer membrane [8]. An alternative pathway leading to CO intermediate for the methanol or formic acid oxidation results in the poisoning of the active Pt surfaces with covalent CO bonding. To avoid this situation, two ways of improvement are considered usually to enhance the Pt activity with catalytic performance: (1) synthesis of the Pt-M bimetallic electrocatalysts and (2) addition of the carbon materials as support [9–11].

The carbon materials (nanoscale active carbon, carbon nanotubes, carbon fibers, and graphene) are used as support to the metal nanoparticles in order to enhance the electrochemical catalytic activities [12–17]. Graphene is one of the most eye-catching materials for researchers in a variety of fields because of its high conductivity and superior mechanical/electrochemical property [18–23]. However, with different methods in synthesized graphenes, these materials are produced with different properties [24–29]. The drawbacks for graphene are its hydrophobicity and the aggregation behavior, which have limited the wet processes to a great extent. Some research groups were working on modification of the graphene surface by ionic liquids and/or polymeric materials [30, 31]. Poly(diallyldimethylammonium) chloride, abbreviated as PDDA, was introduced in this study because the functional groups and the non-covalent interactions of polymer with the graphene surface maintain the nice property of graphene and in addition enhance the electrocatalytic capability [32, 33]. Herein, we used a hydrothermal method to produce the Au nanoparticles and the bimetallic PtAu nanoparticles on PDDA-modified graphene sheets (Au/PPDA-G and PtAu/PDDA-G, respectively) and would use these novel nanocomposites to make formic acid oxidation processes.

## Methods

### PDDA-modified Graphene (PDDA-G)

The preparation of graphene followed the modified Hummer’s method. Into a 250-mL round-bottomed flask held in iced bath, it was introduced with concentrated sulfuric acid (25 mL) then fumed with nitric acid (10 mL in droplets) for 15 min. With iced bath, graphite powder (1 g) (Sigma-Aldrich, Ltd) was introduced into the round-bottomed flask under vigorous stirring. After being well mixed, the solution was added with potassium permanganate (22 g) for 30 min before the iced bath was removed. The solution was then stirred at room temperature for 96 h. Suitable (300 ~ 500 mL) amounts of doubly ion-exchanged (D.I.) water were added to the product mixture while kept in ice bath then the solution was centrifuged for removal of the supernatant. This procedure was performed for three times. The collected precipitates were rinsed with methanol for three times. The muddy-like solid was dried at 80 °C for 12 h to yield the graphite oxide.

In a 100-mL Teflon-lined autoclave with 65 mL D.I. water, 193.8 mg of graphite oxide and 2.54 mL of PDDA (35 wt. %) were added. Then, the autoclave was heated in an oven at 90 °C for 5 h then cooled to room temperature, before removal of the upper clear solution and the solid materials washed with D.I. water for three times. The muddy-like remains were dried at 80 °C overnight. The PDDA-G was then ready to proceed to the deposition of nanoparticles.

### Nanoparticles Deposition on PDDA-G

Fifty milliliters of D.I. water, 100 mg PDDA-G, 131.1 mg H_2_AuCl_4_ (Sigma-Aldrich, Ltd), and 8 mL hydrazine monohydrate (98 %, Sigma-Aldrich, Ltd) were added and then the mixture was sonicated for 30 min before the mixture was transferred into a 100-mL Teflon-lined autoclave apparatus and heated at 90 °C for 24 h. The composite of Au nanoparticles on PDDA-modified graphene (Au/PDDA-G) as a solution was centrifuged three times with D.I. water followed with removal of supernatants. The residuals were dried under vacuum at 90 °C for 24 h and then collected.

The composite of bimetallic PtAu nanoparticles on PDDA-modified graphene (PtAu/PDDA-G) was obtained in a similar way by mixing 99 mg PDDA-modified graphene, 215.3 mg K_2_PtCl_6_ (~0.456 mmol), and 133 mg H_2_AuCl_4_ (~0.456 mmol) with 50 mL D.I. water and sonificated for 30 min, followed by addition of 10 mL hydrazine monohydrate (80 %) and sonificated further for 10 min. After that, the mixture was transferred to a 100-mL Teflon-lined autoclave apparatus and heated at 90 °C for 24 h. The collected PtAu/PDDA-G solutions were centrifuged three times with D.I. water followed with removal of supernatants. The residuals were dried under vacuum at 90 °C for 24 h and then collected. The cubic Pt/G was synthesized in the previous method [33].

### X-ray Diffraction (XRD) Characterization

XRD patterns of nanocomposites were examined by Bruker D8 with CuKα X-ray source. The average crystalline sizes of Au/PDDA-G and Pt Au/PDDA-G were estimated by Debye-Scherrer equation (1)

1 $$ D=K\lambda /\beta \cos \theta $$

where *D* is the average crystalline sizes in nanometers, *K* is shape factor (0.94 for this case), *λ* is the wavelength of X-rays, Cu*Kα* = 0.154 nm is the wavelength (nm), and *β* is full width at half maximum of peaks at 2*θ* in the pattern [34].

### Transmission Electron Microscopy (TEM) Analysis

The TEM analysis employed a JOEL 2100 LaB6 light source, 200 keV, and equipped with EDX. The TEM samples were prepared with ethanol dispersion dropped on the grid Cu net for analysis. The selected area electron diffraction (SAED) was employed to reveal Au/PDDA-G and PtAu/PDDA-G electronic diffraction pattern and the lattice *d*-spacing.

### Thermal Gravimetric Analysis (TGA)

TGA was examined with the Perkin Elmer Pyris 1 TGA from room temperature to 800 °C at a heating rate of 10 °C min^−1^ with about 5 mg of sample loading on the platinum plate.

### X-ray Photoelectron Spectroscopic (XPS) Analysis

The XPS analysis was measured by Physical Electronics PHI Quantra XPS microprobe equipped with an Al *Kα* monochromatic X-ray source (1486.6 eV, 45° incident angle).

### Electrochemical Analysis

The electrochemical catalysts (11.3 mg Au/PDDA-G and 13.0 mg PtAu/PDDA-G, separately) were dispersed in 5 mL D.I. water with super sonification for 30 min. The electrochemical electrode was prepared by dropping 5 uL of the above dispersions on the glass-carbon electrode (0.0706 cm^2^) with constant-volume pipet and dried at room temperature. The procedure was repeated for three times. The electrochemical properties were recorded with an Autolab PGSTAT30 potentiostat/galvanostat. These specimens were investigated with the cycle voltammetry (CV, voltage scan range from −0.25 to 1.0 V, at a scan rate of 50 mV/s) and electrochemical impedance spectroscopy (EIS, scan frequency from 10 kHz to 100 mHz at potential 0.3 V with 5 mV amplified) [35].

## Results and Discussion

**Fig. 1:**
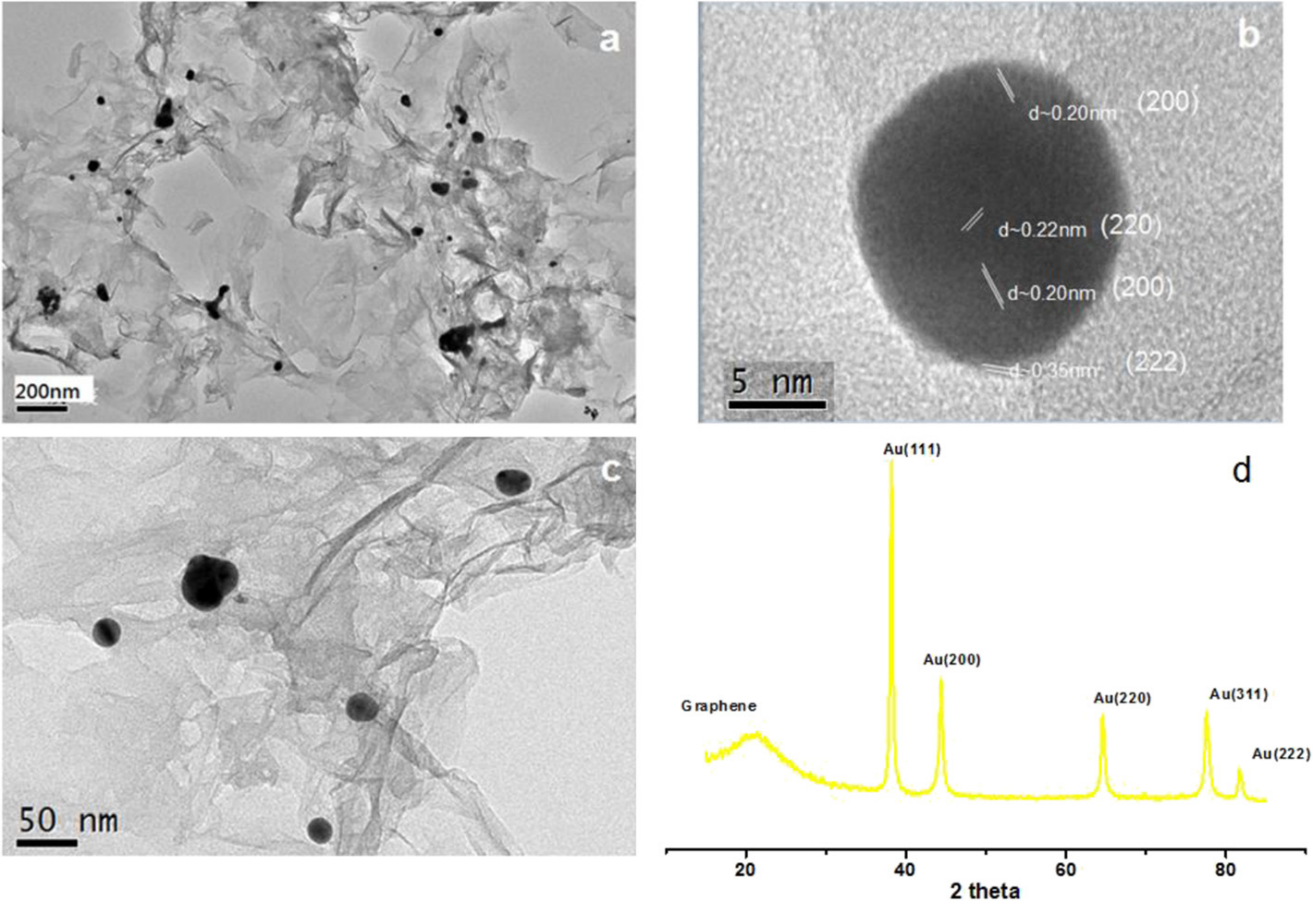
TEM and XRD results of Au/PDDA-G electrocatalyst, at **a** low magnification; **b** high-resolution TEM and lattice image analysis; **c** intermediate magnification; **d** XRD pattern

**Fig. 2:**
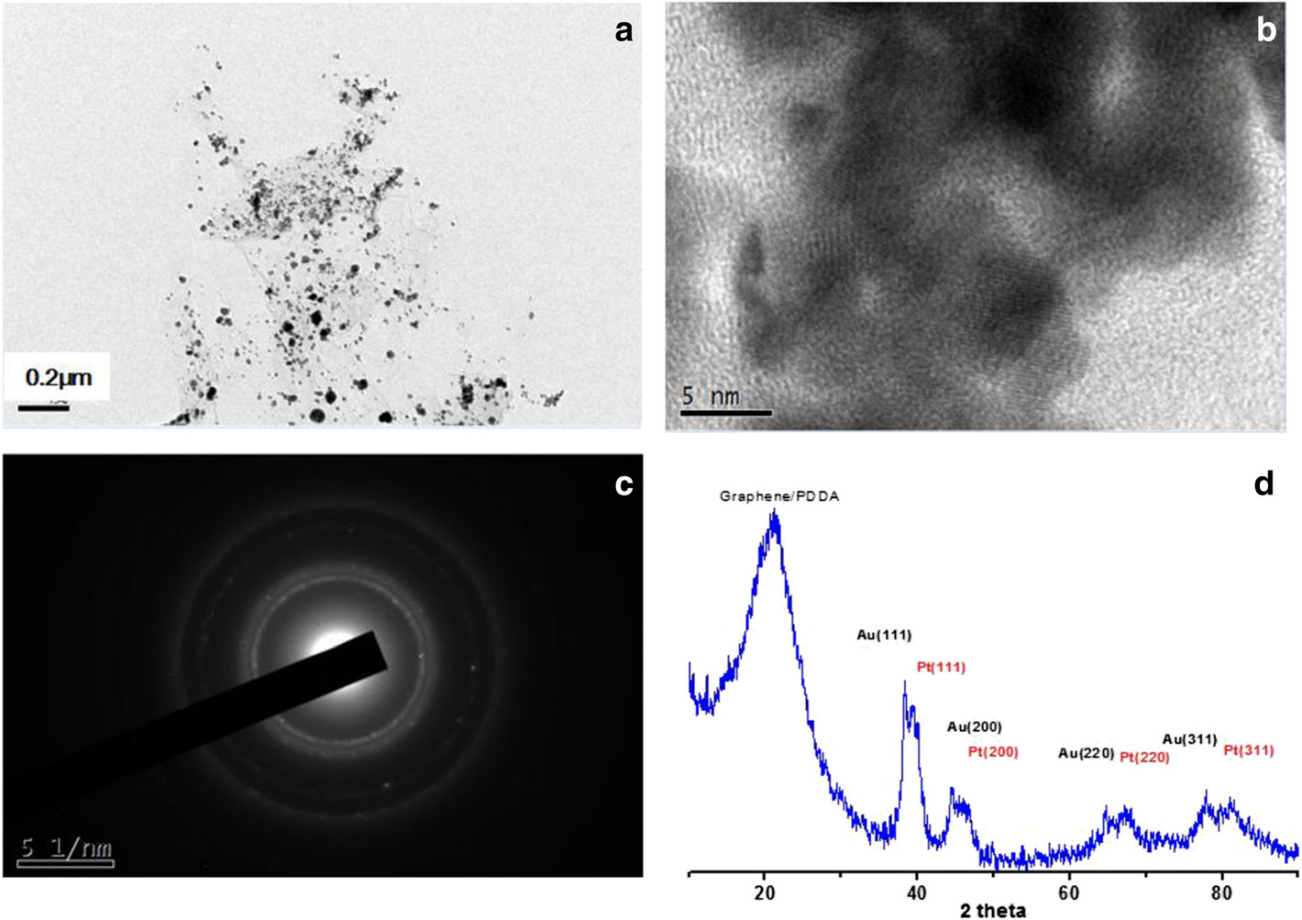
TEM and XRD results of PtAu/PDDA-G electrocatalyst, at **a** low magnification; **b** high-resolution TEM image; **c** SAED pattern; **d** XRD pattern

Au/PDDA-G and PtAu/PDDA-G were synthesized easily in a one-pot process using the hydrothermal method at 90 °C for 24 h. These nanocomposites were with characteristic crystalline structures of the metal nanoparticles. Figure 1 shows the XRD results of the nanocomposite Au/PDDA-G, e.g., 2*θ* of Au plane (111) at 32.23°, (200) at 44.37°, (211) at 64.67°, (311) at 77.52°, and (222) at 81.88°, respectively The bimetallic nanocomposite of PtAu/PDDA-G exhibits 2*θ* of Pt plane (111) at 39.28°, (200) at 46.22°, and (220) at 67.35° in XRD patterns, respectively (Fig. 2). The estimation for the nanoparticle sizes was followed with Debye-Scherrer equation, shown in formula (1). The roughly calculated particle size for Au/PDDA-G is 32.9 nm and PtAu/PDDA-G is 1.8 nm, respectively. Compared to TEM results, the size distributions of the Au nanoparticles and of the bimetallic PtAu on PDDA-modified graphene sheets are 20–50 and 5–10 nm, similar with the XRD results, as shown in Figs. 1a–c and 2a–c, respectively. The PDDA-modified graphene sheet (PDDA-G) shows an XRD peak at 2*θ* = 21.35°, with layer distance seems to be longer than that of pristine graphene sheets (200) at 2*θ* 25°–26° [36], which means that PDDA has immobilized on the graphene sheets.

**Fig. 3:**
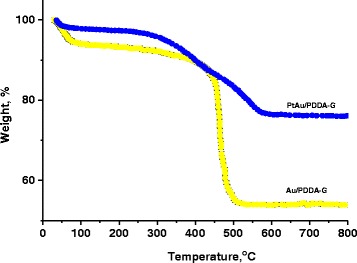
TGA results of Au/PDDA-G and PtAu/PDDA-G electrocatalysts

The thermal analysis (TGA) results in Fig. 3 indicate the metal nanoparticles loading on the PDDA-modified graphene. The PtAu/PDDA-G nanocomposite exhibited weight loss between 350 and 550 °C and the Au/PPDA-G was observed in 450–500 °C. The better the thermal stability attributed to the alloyed PtAu attached on the PDDA-G surface could absorb, the higher the thermal energy of Au/PDDA-G, which increases the temperature of the decomposition. Furthermore, the inorganic content (metal + graphene) of Au/PDDA-G and bimetallic PtAu/PDDA-G is about 54.2 and 76.2 wt. %, respectively. If the content of PDDA and graphene is constant, TGA results show that higher ratios of the bimetallic PtAu nanoparticles were adsorbed on the surface of PDDA-graphene substrate, compared to the Au/PDDA-G system.

**Fig. 4:**
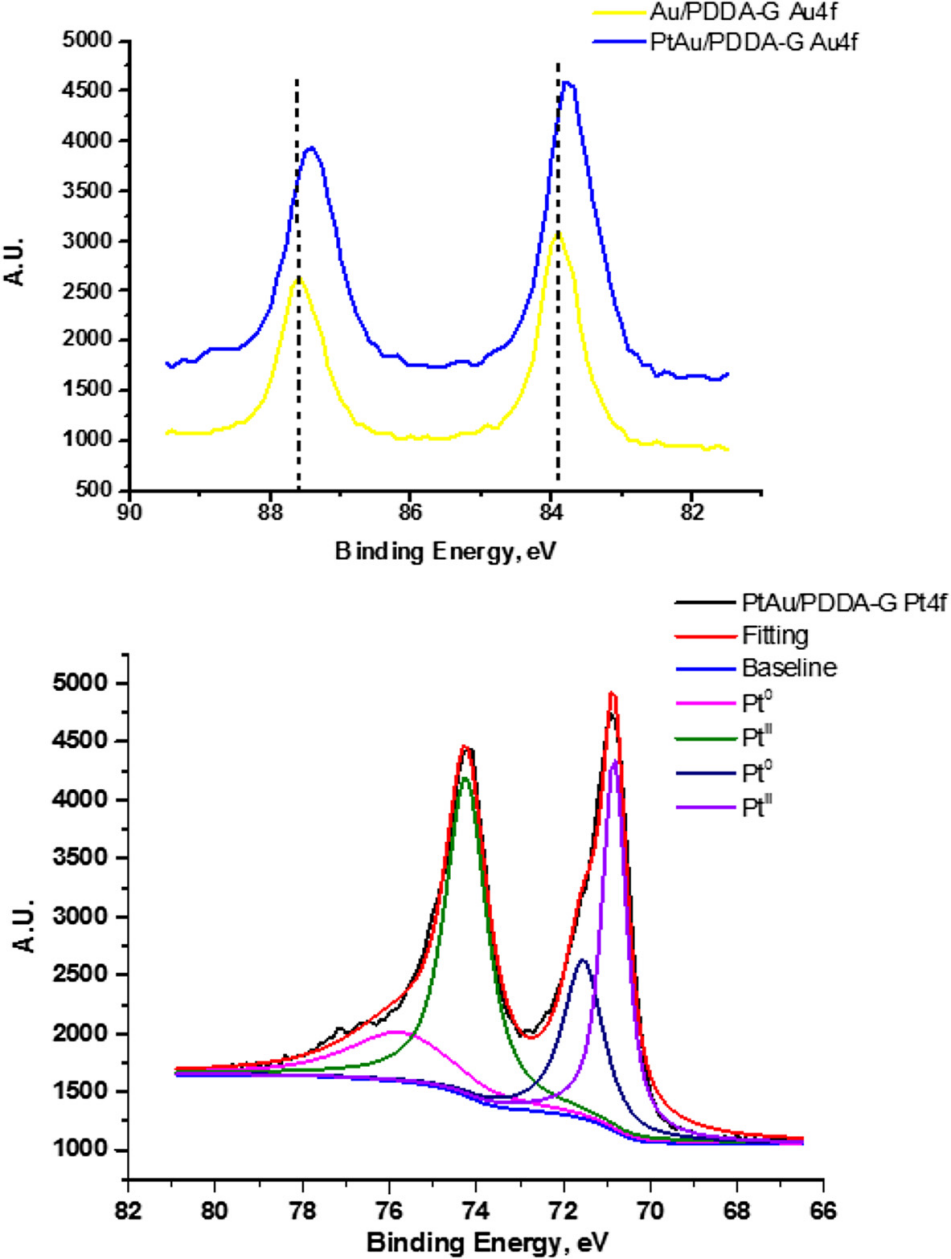
XPS spectra of Au 4f and Pt 4f binding energies in the Au/PDDA-G and PtAu/PDDA-G electrocatalyst

**Fig. 5:**
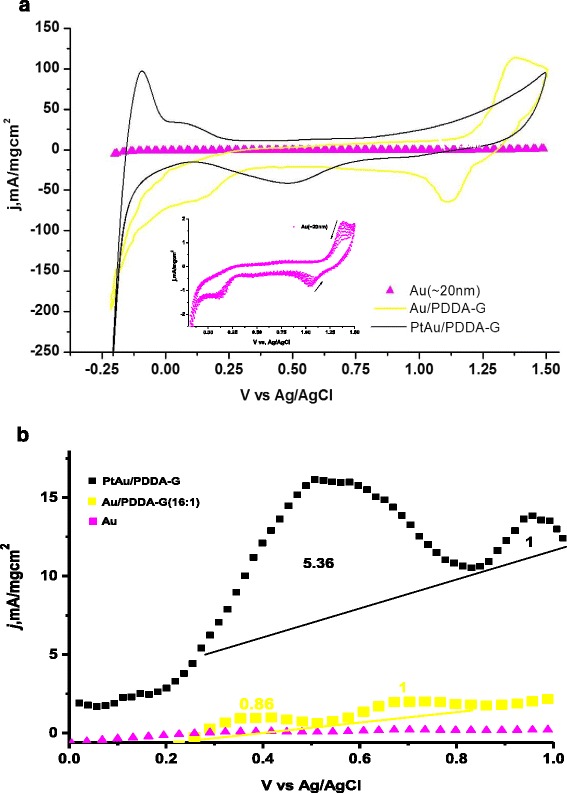
The electrochemical study in aqueous 0.5 M H_2_SO_4_ + 0.5 M HCOOH. **a** The cyclic voltammetric study in 0.5 M aqueous H_2_SO_4_. The *inset* is the same electrical chemical study for Au nanoparticles. **b** The HCOOH oxidation test for three electrocatalysts in 0.0–1.0 V range. The *numbers* are the area ratios with respect to the *dash baselines*. The *inset* is the test for Au/PDDA and Au, respectively

The metal nanoparticles deposited on the PDDA-modified graphene were characterized also with XPS. The Au 4f and Pt 4f binding energies are shown in Table 1, Figs. 4 and 5, respectively. The Au^0^ 4f binding energies are 83.9 ~ 83.7 and 87.7 ~ 87.5 eV in the Au/PDDA-G sample and in the bimetallic PtAu/PDDA-G sample, respectively. The Pt 4f binding energies were curve fitted to resolve the Pt^0^ and Pt^II^ states. The binding energy for Au is shifted by 0.2 eV from Au/PDDA-G to PtAu/PDDA-G, consistent to a PtAu alloy chemical environment. The XPS spectral deconvolution results on C_1s_, O_1s_, and N_1s_ for Au/PDDA-G and PtAu/PDDA-G were shown in Table 1. The N-1s binding energies decrease from PDDA-G, Au/PDDA-G to PtAu/PDDA-G, attributed to metal deposition on the surface of PDDA-G.

**Table 1:** XPS spectral fittings of Au/PDDA-G, PtAu/PDDA-G, and PDDA-G electrocatalysts

XPS PtAu/PDDA-G				
C_1s_	285.7 eV (%)		284.6 eV (%)	
	33.81		66.19	
O_1s_	533.8 eV (%)	532.1 eV (%)	531.1 eV (%)	530.2 eV (%)
	37.95	30.70	21.62	9.63
N_1s_	401.1 eV (%)		399.6 eV (%)	
	58.98		41.02	
Pt_4f_	76.2 eV (%)	74.3 eV (%)	71.4 eV (%)	70.8 eV (%)
	19.26	80.74	50.85	49.15
Au/PDDA-G				
C_1s_	285.5 eV (%)		284.6 eV (%)	
	36.51		63.49	
O_1s_	533.4 eV (%)	532.2 eV (%)	531.3 eV (%)	530.5 eV (%)
	30.80	34.48	21.13	13.59
N_1s_	401.5 eV (%)		399.7 eV (%)	
	41.24		58.72	
PDDA-G				
C_1s_	287.5 eV (%)	285.7 eV (%)	284.6 eV (%)	284.1 eV (%)
	1.96	42.69	18.44	36.91
O_1s_	534.2 eV (%)	532.4 eV (%)	531.8 eV (%)	530.7 eV (%)
	16.31	35.52	23.66	24.51
N_1s_	402.0 eV (%)		401.6 eV (%)	
	54.19		45.81

The Au/PDDA-G and PtAu/PDDA-G were studied electrochemically in aqueous solutions made up of 0.5 M H_2_SO_4_ only and made up of 0.5 M H_2_SO_4_ + 0.5 M HCOOH, for determination of electrocatalytical activities. Figure 5a exhibits the cyclic voltammetric results of Au, Au/PDDA-G, and bimetallic PtAu/PDDA-G. The PtAu/PDDA-G specimen clearly demonstrates the hydrogen redox behavior but not the Au-based nanocomposites. The Au specimen works similarly to the Au/PDDA-G specimen, with preference to oxygen reduction reaction, however, the hydrogen redox abilities do not work similarly to the Pt-based electrocatalysts.

The formic acid oxidation on Pt usually follows the so-called dual pathway [37].

I: Direct dehydrogenation producing CO_2_. (iPI)

2 $$ \mathrm{HCOOH}\to {\mathrm{H}\mathrm{COO}}_{\mathrm{ads}}+{\mathrm{H}}^{+} + {\mathrm{e}}^{-} $$

3 $$ {\mathrm{H}\mathrm{COO}}_{\mathrm{ads}}\to {\mathrm{CO}}_2 + {\mathrm{H}}^{+} + {\mathrm{e}}^{-} $$

II: Dehydration generating CO (poisoning intermediate). (iPII)

4 $$ \mathrm{HCOOH}\to \mathrm{C}\mathrm{O} + {\mathrm{H}}_2\mathrm{O}\to {\mathrm{CO}}_2 + 2{\mathrm{H}}^{+}+2{\mathrm{e}}^{-} $$

The peaks at around 0.5 V (iPI) are attributed to the oxidation of HCOOH to CO_2_; the peaks at about 0.92 V (iPII) are due to the presence of CO on surface of metal catalysts (denoted as CO_ads_, as generated from the dissociative adsorption step, whose intensity indicates the amount of CO_ads_ on the surface of electrocatalysts) [3–7, 24]. A high iPI/iPII ratio (5.36 vs. 0.84) together with a low onset potential (0.20 V vs. 0.25 V) for PtAu/PDDA-G vs. Au/PDDA-G, respectively, indicates that the PtAu/PDDA-G prefers the direct dehydrogenation branch in formic acid oxidation, see Fig. 5b.

**Fig. 6:**
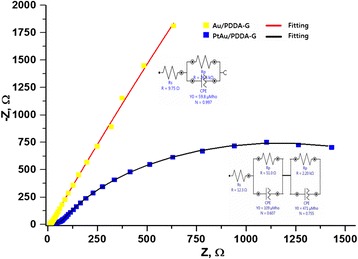
EIS and fitting results for Au/PDDA-G and PtAu/PDDA-G electrocatalysts. The *upper* and *lower insertions* depict equivalent circuit simulations for Au/PDDA-G and PtAu/PDDA-G electrocatalysts, respectively

The electrochemical impedance spectroscopy study at constant 0.3 V revealed the resistance of HCOOH oxidation for the Au/PDDA-G and for the PtAu/PDDA-G nanocomposites. The Nyguist plots are shown in Fig. 6. The electrochemical impedance of Au/PDDA-G was apparently much greater than that of the PtAu/PDDA-G, both being with PDDA-modified graphene sheets as support. The HCOOH oxidation utilizing Au/PDDA-G has very low activity and very high resistance when compared to that utilizing PtAu/PDDA-G (Figs. 5 and 6). The equivalent circuit diagrams for Au/PDDA-G and for PtAu/PDDA-G were shown in Fig. 6, applying the forms of *R*_*s*_(*R*_*p*_*C*) and *R*_*s*_(*R*_*p1*_*C*_*1*_)(*R*_*p2*_*C*_*1*_), respectively, where *R*_*s*_ is for the resistance of electrode, *R*_*p*_ is for the chemical reaction/electron resistance, and *C* is for the electrochemical capacitance attributed to the double layers or interfaces. The *R*_*s*_ of Au/PDDA-G is 9.75 ohm, smaller than that of PtAu/PDDA-G, 12.3 ohm. Thus, the resistance of electrode for Au/PDDA-G is smaller than that for PtAu/PDDA-G. On the other hand, the chemical reaction/electron resistance is reverse in order: *R*_*p*1_ (51.0 ohm) and *R*_*p*2_ (22.2 kohm) for PtAu/PDDA are both smaller than *R*_*p*_ (26.6 kohm) for Au/PDDA-G.

**Fig. 7:**
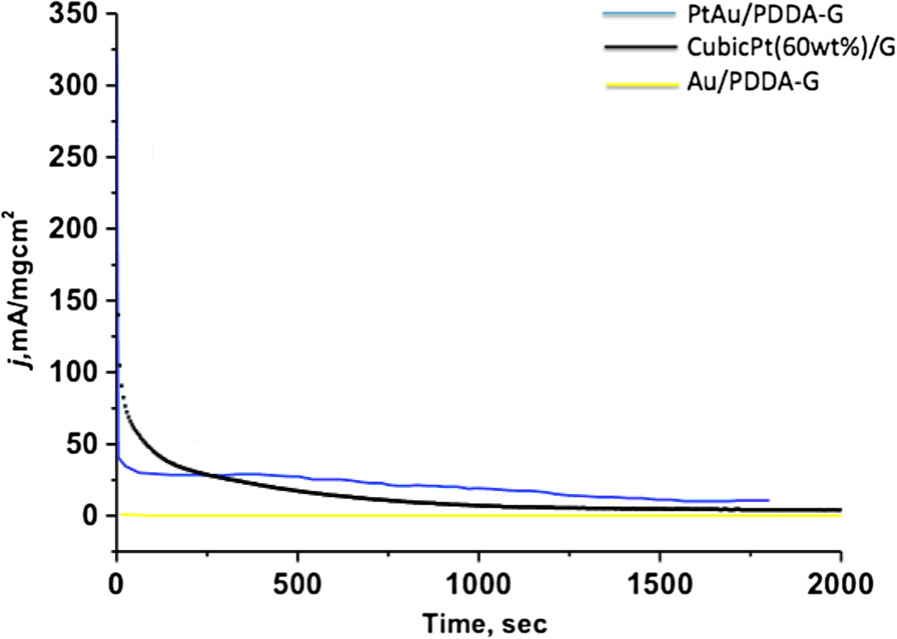
The electrochemical catalysts durability test for formic acid oxidation at the potential 300 mV for about 3000 s. The *blue line* is the result for PtAu/PDDA-G, the *black line* is for cubic Pt (60 wt.%)/G, and the *yellow line* is for Au/PDDA-G

For catalytic HCOOH oxidation activities, the cubic Pt/G was found very active but the activity followed a hyperbolic decay to ~5 mA/mgcm^2^ in 1800 s. The PtAu/PDDA-G was found very active during the initial 10 s and then the activity followed a slow, linear drop to 18 mA/mgcm^2^ after 1800 s. However, the Au/PDDA-G was found to exhibit bad performance on oxidation and bad stability. The long-term stability in decreasing order shows as follows: PtAu/PDDA-G > cubic Pt/G > Au/PDDA-G, as shown in Fig. 7.

## Conclusions

We have successfully used a one-pot hydrothermal method to synthesize the Au/PDDA-G and the bimetallic PtAu/PDDA-G nanocomposites for formic acid oxidation. TEM, XRD, and XPS results illustrate that the Au (20–50 nm) and bimetallic PtAu (5–10 nm) nanoparticle could grow well on the graphene sheets. From the oxidation activity test, PtAu/PDDA-G exhibits the highest iPI/iPII ratio, represents the best capability in anti-CO poisoning, and hence displays the greatest stability in long-term operation. The novel PtAu/PDDA-G is potential to be applied in the electrocatalyst for formic acid oxidation.

## References

[1] Yu X, Pickup PG (2008). Recent advances in direct formic acid fuel cells (DAFAFC). J Power Sources.

[2] Leng J, Wang WM, Lu LM, Bai L, Qiu XL (2014). DNA-templated synthesis of PtAu bimetallic nanoparticle/graphene nanocomposites and their application in glucose biosensor. Nanoscale Res Lett.

[3] Ha S, Larsen R, Masel RI (2005). Characterization and formic acid oxidation studies of PtAu nanoparticles. J Power Sources.

[4] Saipaya S, Srisombat L, Wongtap P, Sarakonsri T (2014). Characterization and formic acid oxidation studies of PtAu nanoparticles. J Nanosci Nanotech.

[5] Li C, Yamauchi Y (2013). Facile solution synthesis of Ag@Pt core-shell nanoparticles with dendritic Pt shell. Phys Chem Chem Phys.

[6] Ataee-Esfahani H, Imura M, Yamauchi Y (2013). All metal mesoporous nanocolloids: solution-phase synthesis of core-shell Pd@Pt nanoparticles with a designed concave surface. Angew Chem Int Ed.

[7] Ataee-Esfahani H, Nemoto Y, Imura M, Yamauchi Y (2012). Facile synthesis of nanoporous Pt-Ru alloy spheres with various compositions toward highly active electrocatalysts. Chem Asian J.

[8] N. Kristian, Y. S. Yan, X.Wang. Highly Efficient Submonolayer Pt-decorated Au Nano-catalysts for Formic Acid Oxidation. Chem Commun. 2008; 353–355. 10.1039/b714230g 18399205

[9] Antolini E, Salgado JRC, Gonzales ER (2006). The stability of Pt-M (M = first row transition metal) alloy catalysts and its effect on the activity in low temperature fuel cells: a literature review and tests on a Pt-Co catalyst. J Power Sources.

[10] Qian Y, Wen W, Adcock PA, Jiang Z, Hakim N, Saha MS (2008). PtM/C catalyst prepared using reverse micelle method for oxygen reduction reaction in PEM fuel cells. J Phys Chem.

[11] Yao KS, Chen YC, Chao CH, Wang WF, Lien SY, Shih HC (2010). Thin solid films: electrical enhancement of DMFC by Pt-M/C catalyst-assisted PVD. Thin Solid Films.

[12] Uhm S, Lee HJ, Kwon Y, Lee J (2008). A stable and cost-effective anode catalyst for formic acid fuel cells. Angew Chem.

[13] Yu XW, Pickup PG (2008). Preparation of Pd/C catalyst for formic acid oxidation using a novel colloid method. J Power Sources.

[14] Huang YJ, Zhou XC, Liao JH, Liu CP, Lu TH, Xing W (2008). Preparation of Pd/C catalyst for formic acid oxidation using a novel colloid method. Electrochem Commun.

[15] Tian N, Zhou ZY, Sun SG, Ding Y, Wang ZL (2007). Synthesis of tetrahexahedral platinum nanocrystals with high-index facets and high electro-oxidation activity. Science.

[16] Mazumder V, Sun SH (2009). Oleyamine-mediated synthesis of Pd nanoparticles for catalytic formic acid oxidation. J Am Chem Soc.

[17] Chen YX, Heinen M, Jusys Z, Behm RB (2006). Kinetic and mechanism of the electrooxidation of formic acid—spectroelectrochemical studies in a flow cell. Angew Chem.

[18] Luo J, Wang L, Mott D, Njoki PN, Lin Y, He T (2008). Core/shell nanoparticles as electrocatalysts for fuel cell reactions. Adv Mater.

[19] Lin YH, Cui XL, Yen C, Wai CM (2005). Pt/carbon nanofiber nanocomposites as electrocatalysts for direct methanol fuel cells: prominent effects of carbon nanostructures. J Phys Chem B.

[20] Xu CW, Wang H, Shen PK, Jiang SP (2007). Highly ordered Pd nanowire arrays as effective electrocatalysts for ethanol oxidation in direct alcohol fuel cells. Adv Mater.

[21] Xu JB, Zhao TS, Liang ZX, Zhu LD (2008). Facile preparation of AuPt alloy nanoparticles from organometallic complex precursor. Chem Mater.

[22] Zhou WP, Lewera A, Larsen R, Masel RI, Bagus PS, Wieckowski A (2006). Size effects in electronic and catalytic properties of unsupported palladium nanoparticles in electrooxidation of formic acid. J Phys Chem B.

[23] Hoshi N, Kida K, Nakamura M, Nakada M, Osada K (2006). Structural effects of electrochemical oxidation of formic acid on single crystal electrodes of palladium. J Phys Chem B.

[24] Solla-Gullon J, Montiel V, Aldaz A, Clavilier J (2002). Electrochemical and electrocatalytic behaviour of platinum-palladium nanoparticles alloys. Electrochem Commun.

[25] Babu PK, Kim HS, Chung JH, Oldfield E, Wieckowski A (2004). Bonding and motional aspects of CO adsorbed on the surface of Pt nanoparticles decorated with Pd. J Phys Chem B.

[26] Park S, Xie Y, Weaver MJ (2002). Electrocatalytic pathway on carbon-supported platinum nanoparticles: comparison of particle-size-dependent rates of methanol, formic acid, and formaldehyde electrooxidation. Langmuir.

[27] Zhang S, Shao YY, Yin GP, Lin YH (2010). Facile synthesis of PtAu alloy nanoparticles with high activity for formic acid oxidation. J Power Sources.

[28] Peng Z, Yang H (2009). PtAu bimetallic heteronanostructures made by post-synthesis modification of Pt-Au nanoparticles. Nano Res.

[29] Lim B, Jiang MJ, Camargo PHC, Cho EC, Tao J, Lu XM (2009). Pd-Pt bimetallic nanodendrites with high activity for oxygen reduction. Science.

[30] Zhang J, Sasaki K, Sutter E, Adzic RR (2007). Stabilization of platinum oxygen-reduction electrocatalysts using gold clusters. Science.

[31] Lee HJ, Habas SE, Somorjai GA, Yang PD (2008). Localizes Pd overgrowth on cubic Pt nanocrystals for enhanced electrocatalytic oxidation of formic acid. J Am Chem Soc.

[32] Maiyalagan T, Karthikeyan S (2013). Film-pore modeling for sorption of Azo dye on to exfoliated graphitic nanoplatelets. Indian J Chem Technol.

[33] Maiyalagan T, Wang X, Manthiram A (2014). Highly active Pd and Pd-Au nanoparticles supported on functionalized graphene nanoplatelets for enhanced formic acid oxidation. RCS Adv.

[34] Liu TY, Huang LY, Hu SH, Yang MC, Chen SY (2007). Core–shell magnetic nanoparticles of heparin conjugate as recycling anticoagulants. J Biomed Nanotechnol.

[35] Yung TY, Li JY, Liu LK (2013). Graphene nanocomposite with platinum nanoparticles for utilization in methanol oxidation reaction. Sci Technol Adv Mater.

[36] Godinez-Salomón F, Arce-Estrada E, Hallen-López M (2012). Electrochemical study of the Pt nanoparticles size effect in the formic acid oxidation. Int J Electrochem Sci.

[37] Capon A, Parsons R (1973). The oxidation of formic acid on noble metal electrodes: II. A comparison of the behaviour of pure electrodes. J Electroanal Chem.

